# Identification and Characterization of a *Glyoxalase I* Gene in a Rapeseed Cultivar with Seed Thermotolerance

**DOI:** 10.3389/fpls.2016.00150

**Published:** 2016-02-16

**Authors:** Guixin Yan, Xiaodan Lv, Guizhen Gao, Feng Li, Jun Li, Jiangwei Qiao, Kun Xu, Biyun Chen, Limin Wang, Xin Xiao, Xiaoming Wu

**Affiliations:** Key Laboratory of Biology and Genetic Improvement of Oil Crops, Ministry of Agriculture, Oil Crops Research Institute of the Chinese Academy of Agricultural SciencesWuhan, China

**Keywords:** *Glyoxalase I* gene, seed thermotolerance, proteomics, *Brassica napus* L., yeast

## Abstract

Glyoxalase I (GLYI) is a ubiquitous enzyme in all organisms that catalyzes the conversion of the potent cytotoxin methylglyoxal to S-D-lactoylglutathione. Although many reports suggest the importance of GLYI in the plant response to stress, its function in seeds requires further study. Here, we identified a heat-induced GLYI from *Brassica napus* seeds, BnGLYI, using a comparative proteomics approach. Two-dimensional gel analyses revealed that BnGLYI protein expression upon heat treatment was significantly elevated in thermotolerant seeds but was diminished in heat-sensitive seeds. The *BnGLYI-2* and *BnGLYI-3* genes from the heat-sensitive and thermotolerant cultivars, respectively, were characterized, and analyzed. Only two amino acid residue variations were found between the amino acid sequences of the two genes. Moreover, overexpressing *BnGLYI-3* in yeast cells enhanced tolerance to heat and cold stress and significantly increased GLYI activity compared to overexpressing *BnGLYI-2*. In addition, *BnGLYI-3* transformants showed enhanced superoxide dismutase activities under heat and cold treatment, whereas these activities were diminished for *BnGLYI-2* transformants. Taken together, these results indicate that overexpression of the *BnGLYI-3* gene imparts thermotolerance and cold tolerance in yeast and that the variations in BnGLYI-3 may play an important role in the responses to temperature stresses.

## Introduction

Seeds not only provide the majority of the human food supply but are also an important supply of nutrients in animal husbandry and industrial feedstock; high-quality seeds are of great significance for socioeconomic development (Janmohammadi et al., 2008). Seed quality is determined by factors such as the rate and uniformity of germination, vigor, and storability of a seed lot. Biotic and abiotic factors affect seed vigor, germination, and storability. High temperature is one abiotic factor often incurred in seed storage, which may reduce seed vigor and decelerate or completely inhibit seed germination (Wahid et al., 2007). Tests using artificial aging induced by high temperature are therefore often used to evaluate seed storability (Janmohammadi et al., 2008). In addition, heat stress is a major factor that affects plant development (Hall, 1992; Marcum, 1998; Howarth, 2005). Heat stress, usually in combination with drought stress or other stresses, causes extensive worldwide agricultural losses (Suzuki and Mittler, 2006). Although many studies have investigated the molecular factors related to thermotolerance in plants (Wahid et al., 2007), few studies in seeds have been reported.

Several major tolerance mechanisms, including the activities of ion transporters, osmoprotectants, free-radical scavengers, late embryogenesis abundant proteins, and factors involved in signaling cascades and transcriptional control, are essential for helping a seed counteract damage induced by harsh temperatures (Wang et al., 2004). Previous studies examining seed antioxidant defense have shown that ROS can accumulate due to disruption of the mitochondrial electron transport chain (Leprince et al., 1994) in response to recommencement of metabolism during seed aging. An increasing number of HSPs/chaperones that interact with heat stress-response mechanisms have been identified (Wang et al., 2004). Transgenic *Arabidopsis* seeds overexpressing *NnHSP17.5* in sacred lotus (*Nelumbo nucifera* Gaertn) exhibited enhanced resistance to accelerated aging treatment (Zhou et al., 2012). Furthermore, other heat-responsive genes have been identified. Recently, a heat-induced *NnANN1* gene was identified in *N. nucifera* seeds and was demonstrated to play an important role in seed thermotolerance (Chu et al., 2012). However, the molecular basis underlying these mechanisms is currently unclear. Considering that the cellular response to abiotic stresses is a multigenic trait resulting from multiple complex metabolic reactions, additional candidate genes contributing to heat tolerance must be explored.

The glyoxalases have been studied for many years in animals and have been proposed to be involved in the regulation of cell division and proliferation, tumor growth, microtubule assembly, and the protection of cells against oxoaldehyde toxicity (Thornalley, 1990). The glyoxalase system is important for detoxification of MG, a cytotoxic metabolite that is primarily produced as a result of carbohydrate and lipid metabolism. Two metalloenzymes, glyoxalase I (GLYI), and GLYII, constitute the glyoxalase system and catalyze a two-step reaction (Thornalley, 1990). First, GLYI catalyzes the conversion of MG to S-D-lactoylglutathione (S-LG) in the presence of GSH. Second, S-LG is hydrolyzed to D-lactate and GSH by GLYII. In this reaction, GLYI is considered as the primary rate-limiting enzyme (Ferguson et al., 1998). However, only a few *GLYI* genes have been cloned in plants, including *Lycopersicon esculentum*, *Brassica juncea*, *B. oleracea*, *Glycine max*, *Sporobolus stapfianus*, *Triticum aestivum*, *Thlaspi caerulescens*, *Vigna radiata*, and *Oryza sativa* (Espartero et al., 1995; Clugston et al., 1998; Veena et al., 1999; Skipsey et al., 2000; Lin et al., 2010; Hossain et al., 2011; Tuomainen et al., 2011, and Mustafiz et al., 2014). To date, genome projects have revealed that *Arabidopsis thaliana* and *Oryza sativa* contain 11 genes encoding GLYI (Mustafiz et al., 2011).

In plants, GLYI showed higher activity in rapidly dividing cells, such as in cell suspensions (soybean), root tips and seedlings (Paulus et al., 1993; Lin et al., 2010). GLYI activity can also be upregulated under stress conditions and in the presence of developmental cues, and this observation suggests that GLYI plays an important role in the stress response and in development (Ramaswamy et al., 1984; Sethi et al., 1988; Deswal et al., 1993; Yadav et al., 2005a,b; Singla-Pareek et al., 2006; Lin et al., 2010; Hossain et al., 2011). Tomato *GLYI* was upregulated in response to salt stress, and osmotic and phytohormonal stimuli (Espartero et al., 1995). Transgenic tobacco overexpressing the *B. juncea GLYI* gene (*BjGLYI*) showed significantly enhanced tolerance to MG and high salt concentrations compared to non-transgenic plants (Veena et al., 1999; Singla-Pareek et al., 2003). In a recent study, tobacco leaves transgenically overexpressing *TaGLYI* from wheat (*T. aestivum* L.) showed increased tolerance to ZnCl_2_ stress compared to control leaves (Lin et al., 2010). Despite the long-standing interest of plant scientists in the function of *GLYI* in stress responses, the involvement of plant *GLYI* genes in the thermotolerance of seeds has not yet been described.

Rapeseed (canola, oilseed rape; *Brassica napus* L., Brassicaceae family), an important oilseed plants worldwide, often encounters high temperature during storage, which leads to decline or deterioration in seed vigor (Nagel and Börner, 2010). Therefore, it is important to identify thermotolerance-related genes in *B. napus* seeds to improve their storability and thermotolerance. In this study, we analyzed seed tolerance to an extremely high temperature to screen for thermotolerance-related genes. A *B. napus GLYI* gene, *BnGLYI*, was identified by comparative proteomic analysis. Two-dimensional gel analyses showed that the protein level of BnGLYI in thermotolerant seeds was significantly increased in response to heat stress. Heterologous expression of *BnGLYI* in yeast conferred tolerance to severe temperature stress. In conclusion, BnGLYI is a heat-responsive protein that participates in tolerance to heat stress, and the variations in the BnGLYI protein sequence might affect its function. These results shed light on the applicability of a molecular breeding approach to enhance plant thermotolerance and seed storability.

## Materials and Methods

### Heat Stress Assay in Rapeseeds and Collection of Plant Materials

A heat-tolerant and a heat-sensitive *B. napus* cultivar, named 3382 and 2682, respectively, were used in this study. The two cultivars were screened by heat stress treatment from many cultivars of *B. napus* (data unpublished). The heat stress assay was performed using a previously described protocol (Gao et al., 2010; Chu et al., 2012). Briefly, surface-sterilized mature rapeseed seeds were incubated at 37°C for 2 h and then treated at 65°C for 2 h. Immediately after this treatment, the seeds were moved to room temperature and finally incubated at 28°C to assess their ability to germinate. The heat treatments were performed in the dark. Five sets of 100 seeds were used for each genotype. The treated seeds and the control seeds (maintained at 28°C) were simultaneously collected for RNA and protein isolation. The simultaneously harvested seeds were used for either the control or the heat treatment. The leaves from plants grown in a greenhouse were used for DNA extraction according to previously reported methods (Murray and Thompson, 1980).

### Protein Extraction

Protein was extracted from both the treated and control *B. napus* seeds via trichloroacetic acid (TCA)/acetone precipitation as described by Damerval et al. (1986) and Gan et al. (2010) with minor modifications. Briefly, the seeds were ground in a precooled mortar in the presence of liquid nitrogen. The powders were precipitated with 10 volumes of precooled acetone (containing 10% TCA and 0.07% dithiothreitol (DTT)), and the mixtures were incubated at –20°C for 1 h. After centrifuging at 20 000 ×*g* for 30 min and removing the supernatant, the pellet of each sample was rinsed and incubated in ice-cold acetone (containing 0.07% DTT) for 1 h at –20°C. This step was repeated three times. Finally, each pellet was air-dried and resuspended in 500 μl of lysis buffer (7 M urea, 2 M thiourea, 4% 3-[(3-cholamidopropyl) dimethylammonio]-1-propanesulfonate (CHAPS), 1 M phenylmethanesulfonyl fluoride (PMSF), 50 mM DTT, 0.5% Triton X-100, and 0.5% ampholine) and then vortexed for 1 h at room temperature. After centrifugation at 20 000 ×*g* for 30 min, each supernatant was collected in a fresh tube, and the protein concentrations were quantified using Bradford reagent (Aidlab, China) according to the manufacturer’s instructions. The protein samples were stored at –80°C.

### Two-Dimensional Gel Electrophoresis

Two-dimensional gel electrophoresis was performed according to Gan et al. (2010, 2013). For total protein analysis, immobilized pH gradient (IPG) strips of 24 cm in length were rehydrated with 450 μl of rehydration solution for 14 h at 20°C, which was loaded with 1 mg of protein (Gan et al., 2010). Isoelectric focusing (IEF) was performed in an Ettan IPGphor IEF system as described by Gan et al. (2010). After IEF, the IPG strips were equilibrated as described by Gan et al. (2013). The second dimension of separation was performed using a 12.5% SDS-PAGE gel in an Ettan DALTsix system according to Gan et al. (2013). The gel was stained with colloidal Coomassie Brilliant Blue G-250 as previously described (Candiano et al., 2004). The experiment was replicated three times.

### Data Analysis and Protein Identification

Images (300 dpi, 16-bit grayscale pixel depth) were scanned using UMAX Powerlook 2100XL (UMAX, Taipei, China) and were analyzed using Image Master 2D Platinum Software (Version 5.0, GE Healthcare) as described by Hajduch et al. (2007). Protein spots showing a significant difference in intensity (*P* < 0.05) of greater than 1.5-fold were selected and manually excised from the gel for protein identification. Mass spectrometry (MS) analysis of selected protein spots was performed by Shanghai Applied Protein Technology Co., Ltd. (Shanghai, China), using the matrix-assisted laser desorption/ionization time of flight (MALDI-TOF) method (Hajduch et al., 2007). The MS/MS data were searched against the ‘plant’ subset of the NCBI non-redundant sequence database using Mascot^1^ (Matrix Science).

### Isolation of *GLYI* cDNA Clones

To functionally characterize *BnGLYI*, its coding region was amplified according to the sequence of *BjGLYI* (**Table 1**). An aliquot of 5 μl of the cDNA was used as a template to conduct PCR using full-length primers in a PTC-200 Peltier Thermal Cycler (Bio-Rad, USA); the PCR protocol consisted of an initial denaturation at 94°C for 5 min, 30 cycles of denaturation at 94°C for 30 s, annealing at 56°C for 30 s, and extension at 72°C for 1 min, and a final extension of 10 min at 72°C. The desired PCR products were cloned into the pEASY-T1 vector (TransGen Biotech, China), and clones were selected on plates supplemented with IPTG, X-gal, and ampicillin. The clones were identified by PCR and then sequenced. Sequence comparisons were performed using BLAST. The *BnGLYI* sequences cloned from *B. napus* 3382 and 2682 were designated as *BnGLYI-3* (KT720495) and *BnGLYI-2* (KT720496), respectively.

**Table 1:** Primer pairs used in the gene-specific amplification of *GLYI* genes.

Primer name	Fragment (bp)	Primers (5′–3′)	Tm (°C)	Used for	Reference
BnGLYF	558 bp	ATGGCGTCGGAAGCGAAGG	67.9	Gene cloning	Veena et al., 1999
BnGLYR		TCAAGCTGCGTTTCCGGCTG	69.1		
BnGLYIJF	558 bp	GGAATTCCACATAATGGCGTCGGAAGCGAAGG	73.5	Gene expression	This study
BnGLYIJR		TT GCGGCCGCTCAAGCTGCGTTTCCGGCTG	75.7		
ACT1-F	131 bp	ATCATGTTCGAGACTTTCAACG	59.2	qRT-PCR in yeast	Zhang et al., 2015
ACT1-R		AATTGGGACAACGTGGGTAA	60.1		
BnGLYIYF	148 bp	CACGTGTCCTGGGAATGTCAT	63.0	qRT-PCR in yeast	This study
BnGLYIYR		ATTGTTGCGGGTCGACCA	63.5		

### Sequence Analysis

Amino acid (AA) sequence alignment was conducted using the DNAMAN program^2^ The conserved domains within the *GLYI* coding nucleotide sequence were searched in the NCBI database^3^

### Quantitative RT-PCR (qRT-PCR) Analysis

Total RNA was extracted from yeast using an RNeasy Mini Kit (QIAGEN, Germany). RNA pellets were dissolved in DEPC-treated water; the presence of RNA was confirmed by agarose gel electrophoresis; and the RNA concentrations were quantified based on the absorbance of the samples at 260 nm. The RNA samples were reverse-transcribed into first-strand cDNA using a ReverTra Ace-α-TM qPCR RT kit (Toyobo, Japan) and stored at –80°C. Expression of the *GLYI* genes in transgenic yeast was evaluated using LightCycler 480 SYBR Green I Master mix and a LightCycler 480II real-time PCR system (Roche, Switzerland). Expression of the *BnGLYI* gene was normalized to that of *Actin* according to the 2 method aaa (Livak and Schmittgen, 2001) using the following formula:

$$relative\,\;\;\;\exp ression\,=2^{-\left[(CT,\;T\,\arg et-CT,\;Actin)sample-(CT,\;T\,\arg et-CT,\;Actin)calibrator\right]},$$

where CT, _Actin_ is the CT value of the reference gene *Actin*, and CT, _target_ is the CT value of the gene under investigation. The value determined by this formula represents the relative expression of a gene in a sample relative to that in a calibrator. The primers and the amplification conditions used are described in **Table 1.** Each sample was examined in triplicate.

### Ectopic Expression of the *BnGLYI* Gene in Yeast

The coding sequence of the *BnGLYI* gene was amplified from cDNA by PCR using pfu DNA polymerase (Tiangen, China). An *Eco*RI site and a *Not*I site (underlined in **Table 1**) were introduced at the 5′-end and the 3′-end of the PCR product, respectively. The *Eco*RI/*Not*I fragment (558 bp) was directly cloned into the pEASY-T1 vector (TransGen, China) and was then sub-cloned into the *Pichia* expression vector pPIC3.5K (Multi-Copy Pichia Expression Kit, Invitrogen, USA) to generate the pPIC3.5K-BnGLYI-2 and pPIC3.5K-BnGLYI-3 plasmids. These plasmids were introduced into *Pichia pastoris* GS115 cells by electroporation (MicroPulser Electroporation Apparatus, Bio-Rad, USA) according to the manufacturer’s instructions for overexpression of the given fusion protein. Transformants were selected by plating cells on synthetic minimal medium agar lacking histidine, and were then confirmed by PCR using gene-specific primers (**Table 1**). The cells transformed with empty vector (pPIC3.5K), pPIC3.5K-BnGLYI-2 and pPIC3.5K-BnGLYI-3 were designated as GS3.5K, GSGLYI-2, and GSGLYI-3, respectively.

Heterologous expression of *BnGLYI* was induced under the transcriptional control of the yeast AOX1 promoter. Expression of *BnGLYI* in *P. pastoris* GS115 was induced according to the instructions of the Multi-Copy Pichia Expression Kit as well as the method described by Wang et al. (2013) with slight modification. The transformant expressing the highest abundance of GLYI was selected and used for further studies. Aliquots (1 ml) of cells were harvested at 24, 48, 72, and 96 h and then centrifuged at maximum speed for 2–3 min at room temperature to collect the cells. Total cellular protein samples were electrophoresed via 12% SDS–PAGE, followed by Coomassie blue staining.

### Assessment of Yeast Resistance to Temperature Stress

Yeast cultured to the exponential phase in a yeast extract peptone dextrose medium (YPD) was inoculated in buffered glycerol complex medium (BMGY). After induction in buffered methanol complex medium (BMMY), the cultured cells were diluted by 1:10, 000. The diluted cells were treated under heat and cold stress according to a previously reported method (Li et al., 2009) with minor modifications. Three aliquots (100 μl each) were treated at 42°C in a water bath for 2.5, 3.5, and 5 h. Simultaneously, three additional aliquots were frozen at –20°C for 10 days (D) and 13 D. The test samples were cultured on a YPD culture plate at 30°C after the treatments. Three aliquots were directly plated on a YPD plate as a control. The survival rate of the treated cells relative to the control cells is expressed as the percentage of colony-forming units (CFU%). The experiments were repeated three times. Tukey’s test was used to analyze all the data, which were presented as the means ± SD (*n* = 3), to compare the survival rate between the yeast cells harboring *BnGLYI-2* and *BnGLYI-3* and the control cells under either normal or stress conditions. A *P* < 0.05 was considered to indicate a significant difference.

### Determination of Enzyme Activities

Glyoxalase I activity in total protein extracts from yeast was measured as described previously (Singla-Pareek et al., 2003; Mustafiz et al., 2010). Each of the measurements was performed using three replicates. The enzymatic production of S-lactoylglutathione (extinction coefficient 3.37 mM^–1^ cm^–1^) was measured at 240 nm at 15 s intervals for 2 min. Specific activity of GLYI is expressed as μmol S-lactoylglutathione formed/hr/mg protein (Junaid et al., 2004). The yeast protein was extracted using Y-PER Yeast Protein Extraction Reagent (Thermo Scientific, USA). Bradford reagent (Aidlab, China) was used to determine the protein concentrations. ImageJ software was used to determine the relative expression levels of BnGLYI-2 and BnGLYI-3.

Superoxide dismutase (SOD) activity was assessed by monitoring the inhibition of the photochemical reduction of nitroblue tetrazolium (NBT) according to the method of Beauchamp and Fridovich (1971). One unit of SOD activity was defined as the amount of enzyme required to cause 50% inhibition of the rate of NBT reduction at 560 nm (Beauchamp and Fridovich, 1971). SOD activity is expressed as units per milligram of protein.

## Results

### Thermotolerance of Oilseed Seeds

**FIGURE 1:**
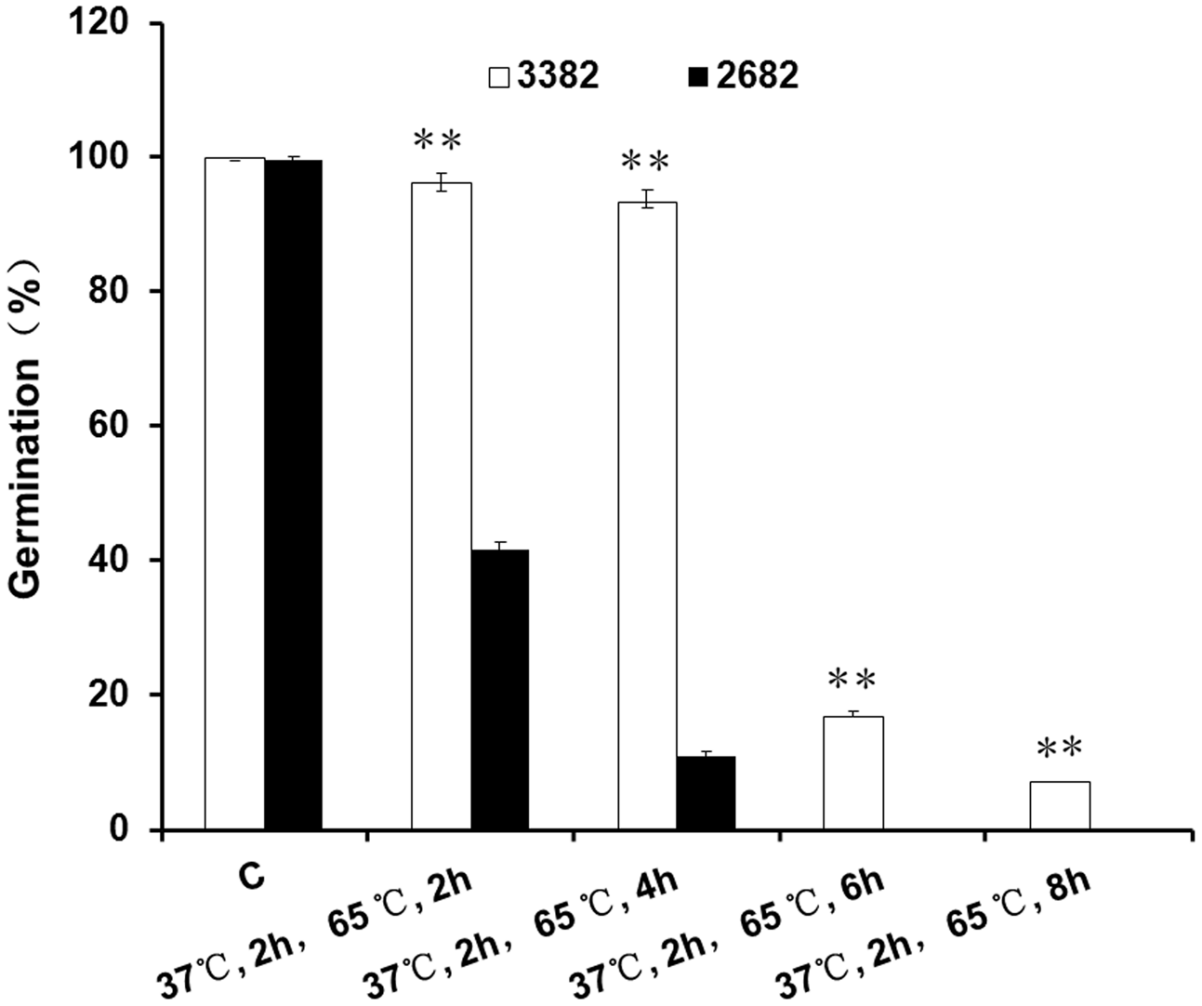
**Seed thermotolerance assays.** Asterisks denote a significant difference compared with 2682 seed germination based on Student’s *t*-test: ^∗∗^*P* < 0.01. The bars represent the means (three replicates) ± SD. C, control.

To demonstrate the basal thermotolerance of oilseeds, mature seeds were treated at high temperature. When treated at 65°C for 2 h, the germination of 3382 seeds was not evidently affected, as only a slight decrease in the germination rate was observed; however, only approximately 40% (41.7 ± 1.2%) of the 2682 seeds remained alive (**Figure 1**). With increasing treatment time, the germination rates of 3382 seeds gradually declined. However, the germination rates of 2682 seeds declined sharply, and up to 100% of the 2682 seeds were dead after 6 h at 65°C. The germination rate of 3382 seeds was significantly higher than that of 2682 seeds under heat treatment.

### GLYI is a Heat-Responsive Protein

**FIGURE 2:**
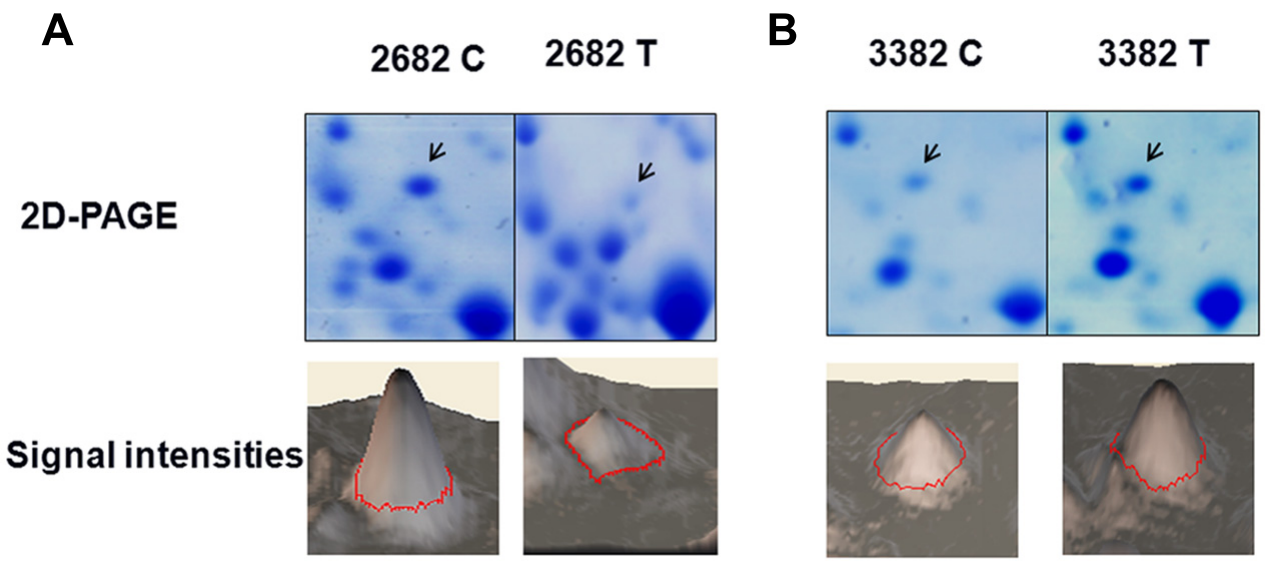
**Glyoxalase I (GLYI) is a heat-responsive protein.** The changes in the level of GLYI protein in response to heat treatment were analyzed by two-dimensional gel electrophoresis. The quantified Coomassie-staining signal intensity of the GLYI spot from each image is, respectively, shown in (**A**, 2682) and (**B**, 3382). C, control, T, treatment.

The objective of this study was to identify novel genes involved in the development of heat tolerance in *B. napus*. To reveal how plant cells respond to heat stress by modulating protein expression, we performed a two-dimensional gel analysis to screen for heat-responsive proteins. One heat-responsive protein was identified as GLYI (**Figures 2A,B**, Supplementary Figures S1 and S2). Nine peptides were matched to the predicted GLYI protein sequence with a MASCOT score of 117 (Supplementary Figure S2).

### Characterization of *GLYI* cDNA Clones from *B. napus*

Based on the sequences of *BjGLYI* (GenBank accession no. Y13239) and *A. thaliana AtGLYI* (AT1G08110.1), full-length cDNAs of *BnGLYI*, encoding proteins sharing 99 and 100% AA identity with the *BjGLYI* sequence, respectively, were cloned from the 3382 and 2682 cultivars. The open reading frame (ORF) of *BnGLYI* cDNA consisted of 558 bp encoding a protein of 185 amino acids. The calculated molecular mass and pI were 20.8 kDa and 5.42, respectively, and these values were consistent with the apparent molecular mass and pI of the target protein spot on the two-dimensional gels.

**FIGURE 3:**
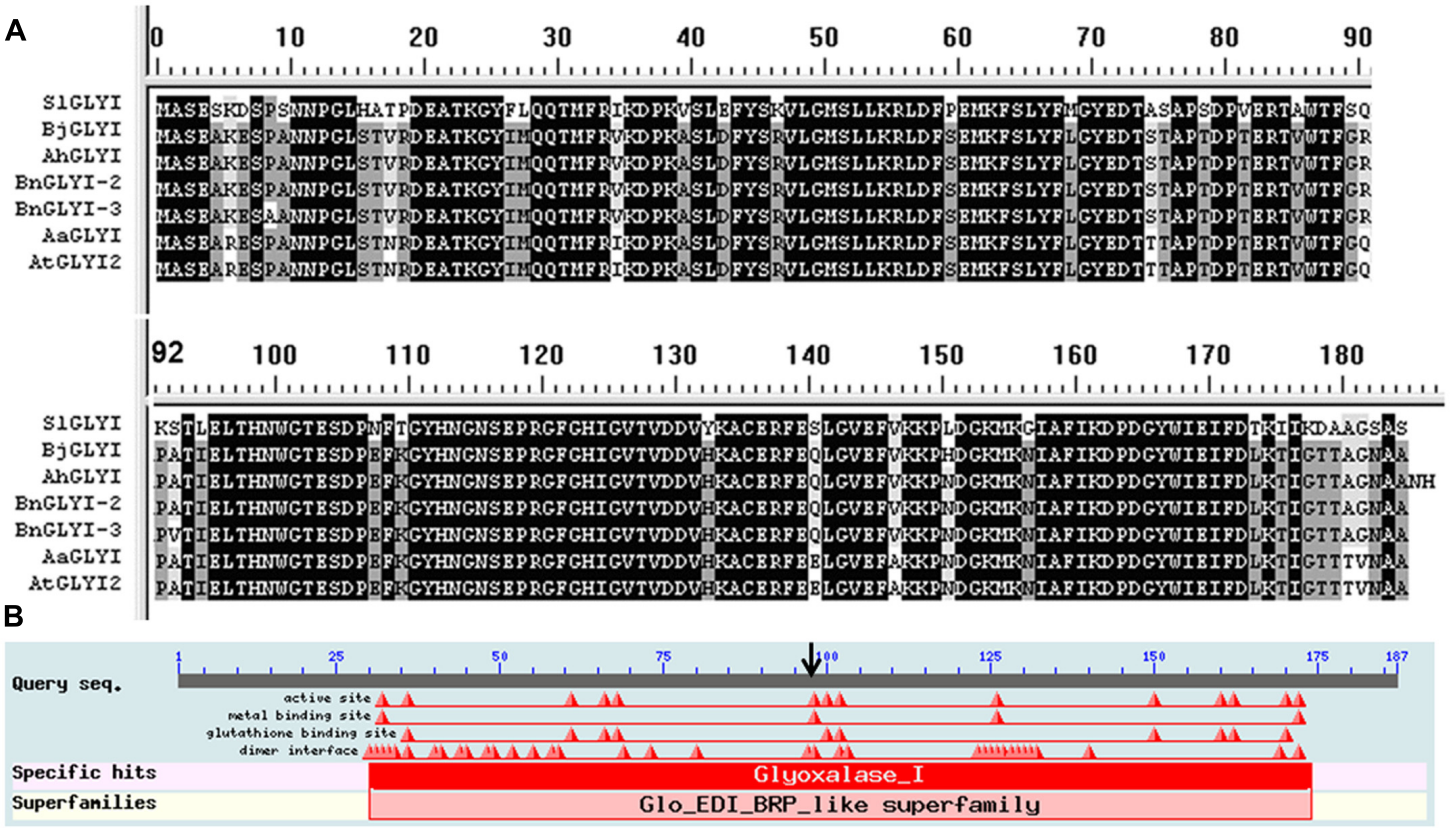
**Sequence alignment and conserved domain analysis of BnGLYI. (A)** Multiple sequence alignment of the deduced AA sequence of the *GLYI* cDNA clone from *B. napus* with previously reported GLYI sequences. The sequences were aligned using the DNAMAN program. SlGLYI: from *Solanum lycopersicum* (Accession no. Z48183), AtGLYI2: from *Arabidopsis thaliana* (Accession no. AT1G08110), BjGLYI: from *Brassica juncea* (Accession no. Y13239), AhGLYI: from *Arachis hypogaea* (Accession no. DQ989209.2), BnGLYI-3: from *B. napus* 3382 (Accession no. KT720495); BnGLYI-2: from *B. napus* 2682 (Accession no. KT720496). **(B)** Conserved domain analysis of BnGLYI. GLYI has a requirement for bound metal ions for catalysis.

Proteins containing a glyoxalase domain (PF00903) and having a putative lactoylglutathione lyase function have been classified as GLYI proteins (Mustafiz et al., 2011). In the two deduced protein sequences, two glyoxalase domains were found (AA 29–171, *E*-value 18.e-25) based on Pfam 23.0^4^, and this result showed that the gene corresponding to both proteins was *BnGLYI*. Multi-alignment analysis of the deduced AA sequences from the *BnGLYI* gene revealed that the *BnGLYI* gene exhibited significantly high similarity to other *GLYI* genes (**Figure 3A**). For example, the BnGLYI-3 protein (from *B. napus* 3382) shared 79, 94, 99, 98, and 99% AA identities with GLYI from *Solanum lycopersicum* (Accession no. Z48183), *A. thaliana* (Accession no. AT1G08110.1), *B. juncea* (Accession no. Y13239), *Arachis hypogaea* (Accession no. DQ989209.2), and *B. napus* 2682 (BnGLYI-2), respectively (**Figure 3A**). These results suggested that this protein has been evolutionarily conserved. AA residues probably involved in metal and GSH binding sites were also conserved among the GLYI sequences (**Figure 3B**). Moreover, we found only two variations (Pro/Ala at position 9 and Ala/Val at position 93) in the AA sequence between BnGLYI-2 and BnGLYI-3. The variation at position 93 may affect the putative metal binding sites and this position may be an active site based on the analysis presented in **Figure 3B.**

### Overexpressing *BnGLYI-3* in *P. pastoris* Enhances Tolerance to Temperature Stresses

**FIGURE 4:**
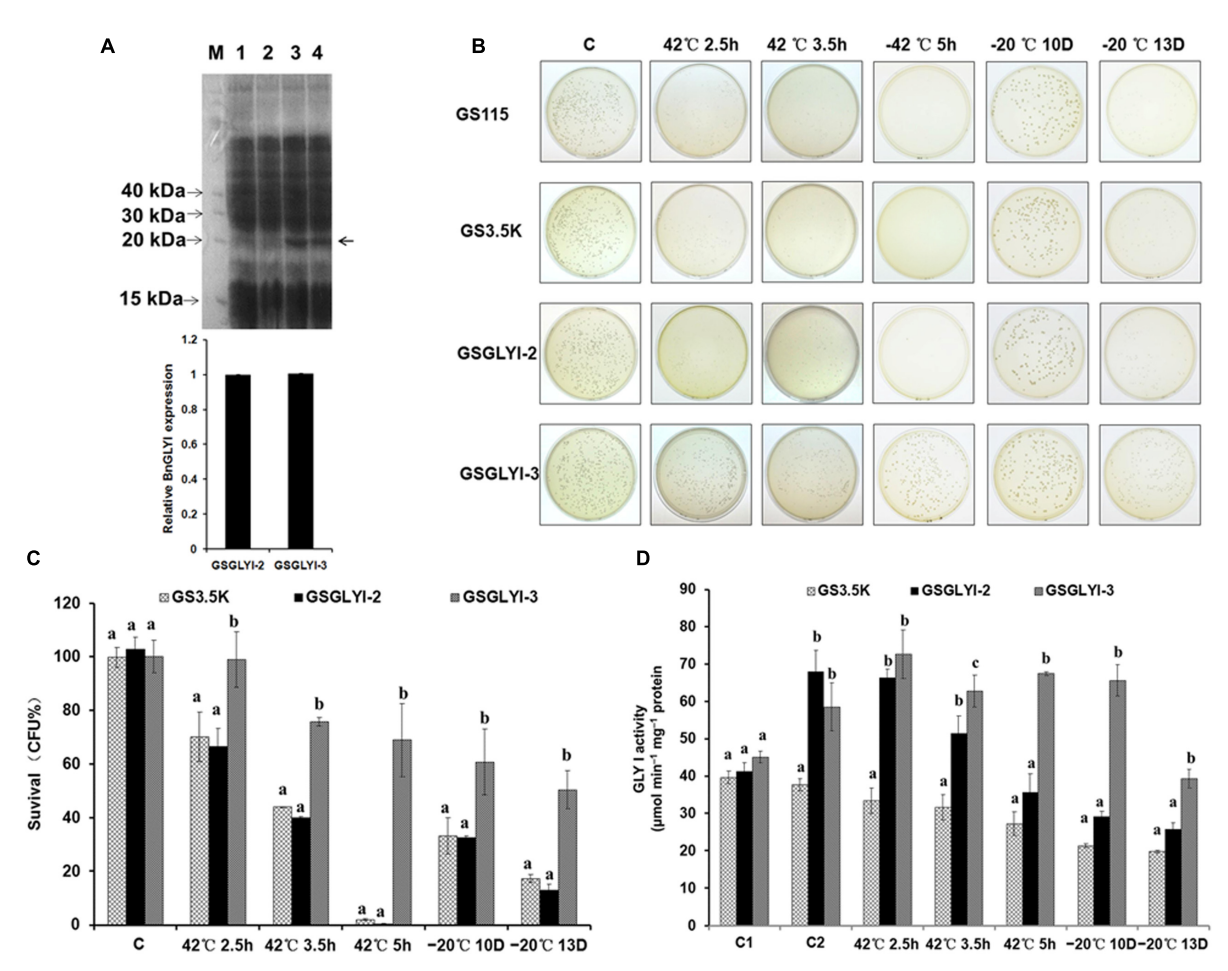
**Analysis of *BnGLYI* expression in *Pichia pastoris*. (A)** SDS-PAGE analysis of *P. pastoris* proteins. M: protein marker (KDa); 1: GS115; 2: GS3.5K; 3: GSGLYI-2; 4: GSGLYI-3. The relative BnGLYI expression levels were calculated after normalization to the total protein content. The expression levels of GSGLYI-2 were normalized to 1.0, and the data were analyzed using ImageJ software. The bars represent the means (three replicates) ± SD. **(B)** Growth of *P. pastoris* cells stressed at 42°C for 2.5, 3.5, and 5 h or stressed at –20°C for 10 D and 13 D. Diluted culture samples were treated at 42°C in a water bath and were then plated on YPD plates that were incubated at 30°C. **(C)** Viability of *P. pastoris* transformants carrying pPIC3.5K-BnGLYI-2 (GSGLYI-2), pPIC3.5K-BnGLYI-3 (GSGLYI-3), or pPIC3.5K (GS3.5K) that were stressed as described in **(B)**. Cell viability is plotted as the percentage of CFUs relative to the number of CFUs in their respective controls. The data are presented as the means ± SD of three independent assays; different letters indicate significant differences (*P* < 0.05) under each stress condition, and values followed by the same letter are not significantly different according to Tukey’s test. **(D)** GLYI activity in crude soluble protein extracted from *P. pastoris* transformed with pPIC3.5K (GS3.5K), pPIC3.5K-BnGLYI-2 (GSGLYI-2), or pPIC3.5K-BnGLYI-3 (GSGLYI-3) under different temperature stress conditions. C, control; C1, non-induced cells cultured under the control conditions; C2, induced cells cultured under the control conditions.

To determine whether the variations between the two BnGLYI protein sequences affected the function of the protein, we overexpressed the two *BnGLYI* genes in *P. pastoris* cells. Each full-length *BnGLYI* coding sequence was subcloned into the pPIC3.5K vector, and the resulting plasmids were transformed into yeast. The qRT-PCR results showed that the transformants harboring *BnGLYI-2* and *BnGLYI-3* displayed significantly increased *BnGLYI* expression, but that *BnGLYI* expression was not detected in the transformants carrying the empty vector or in the wild-type cells (Supplementary Figure S3). In addition, the soluble proteins extracted from *P. pastoris* cells were analyzed by SDS-PAGE. A distinct band with a molecular mass of ^∗^21 kDa was visible in the protein extract obtained from the GSGLYI-2 and GSGLYI-3 cells (**Figure 4A**). Moreover, the GSGLYI-2 and GSGLYI-3 cells exhibited a similar BnGLYI protein level for the same amount of total protein (**Figure 4A**). These results indicated that the two sequence variations did not affect BnGLYI protein stability. Serial dilutions (10^–4^, 10^–5^, 10^–6^) of cell cultures plated on a YPD plate showed that the transformants had similar growth rates to GS3.5K and GS115 cells when incubated at 30°C (data not shown).

A thermotolerance assay was conducted on yeast cells transformed with the recombinant plasmids pPIC3.5k-BnGLY-2 and pPIC3.5k-BnGLY-3, and cells harboring the empty pPIC3.5K vector were used as a control. Compared with the GS3.5K cells, the GSGLYI-3 cells showed increased tolerance to high temperatures; however, the GSGLYI-2 transformant did not show any thermotolerance under heat stress (**Figures 4B,C**). Within the first 2.5 h of heat treatment, loss of cell viability was observed. The survival rates of GS3.5K, GSGLYI-2, and GSGLYI-3 cells were approximately 70.2, 66.7, and 99.1%, respectively. The viability of GSGLYI-3 cells was significantly different from that of GS3.5K and GSGLYI-2 cells (*P* < 0.05). After 3.5 h of heat treatment at 42°C, 40.0 and 75.9% of the cells overexpressing *BnGLYI-3* and *BnGLYI-2*, respectively, had survived. After 5 h of heat treatment at 42°C, 70.1% of the GSGLYI-3 cells remained alive, but most of the GS3.5K and GSGLYI-2 cells were dead (**Figures 4B,C**). Taken together, the survival rate of the *BnGLYI-3*-transformed cells was significantly higher than that of the *BnGLYI-2* and empty vector transformants. However, the survival rate of GSGLYI-2 cells was not significantly different from that of GS3.5K cells.

To investigate the role of the BnGLYI protein in the response to cold shock, the GSGLYI-2 and GSGLYI-3 transformants as well as the empty vector control transformants were treated at –20°C. After being frozen for 10 D, approximately 60% of the GSGLYI-3 cells and 30% of the GSGLYI-2 and GS3.5K cells remained alive. After being frozen for 13 D, clearly more surviving cells (approximately 50%) remained in the GSGLYI-3 cell lines than in the GSGLYI-2 and control GS3.5K cells. Statistical analysis revealed that the survival rate of the GSGLYI-3 cells was 50.5±13.1%, whereas the survival rate of GSGLYI-2 and GS3.5K was approximately 17.4±1.5 and 12.9±2.5%, respectively (**Figures 4B,C**). These results indicated that overexpressing the *BnGLYI-3* gene significantly enhanced the freeze tolerance of the host cells.

Furthermore, we measured GLYI activity in the crude protein samples extracted from GSGLYI-2, GSGLYI-3, and GS3.5K cells. As shown in **Figure 4D**, GS3.5K, GSGLYI-2, and GSGLYI-3 cells exhibited similar GLYI activity under normal conditions without induction. After induction, the GSGLYI-3 and GSGLYI-2 cells displayed approximately 55 and 80% higher activity, respectively, than the GS3.5K cells. GSGLYI-3 cells showed significantly higher GLYI activity than the GS3.5K and GSGLYI-2 cells after treatment at 42°C for 3.5 and 5 h, as well as after treatment at –20°C (**Figure 4D**). GSGLYI-3 cells showed approximately 1.4-fold and 0.9-fold higher GLYI activity than GS3.5K and GSGLYI-2 cells, respectively, after the 5-h treatment. In addition, GSGLYI-3 cells displayed approximately twofold and 1.3-fold higher GLYI activity than GS3.5K and GSGLYI-2 cells, respectively, after 10 D of treatment at –20°C. These results suggested that overexpressing GSGLYI-3 may improve heat and cold stress tolerance in *P. pastoris*.

### SOD Activity in the Transformed Cells

**FIGURE 5:**
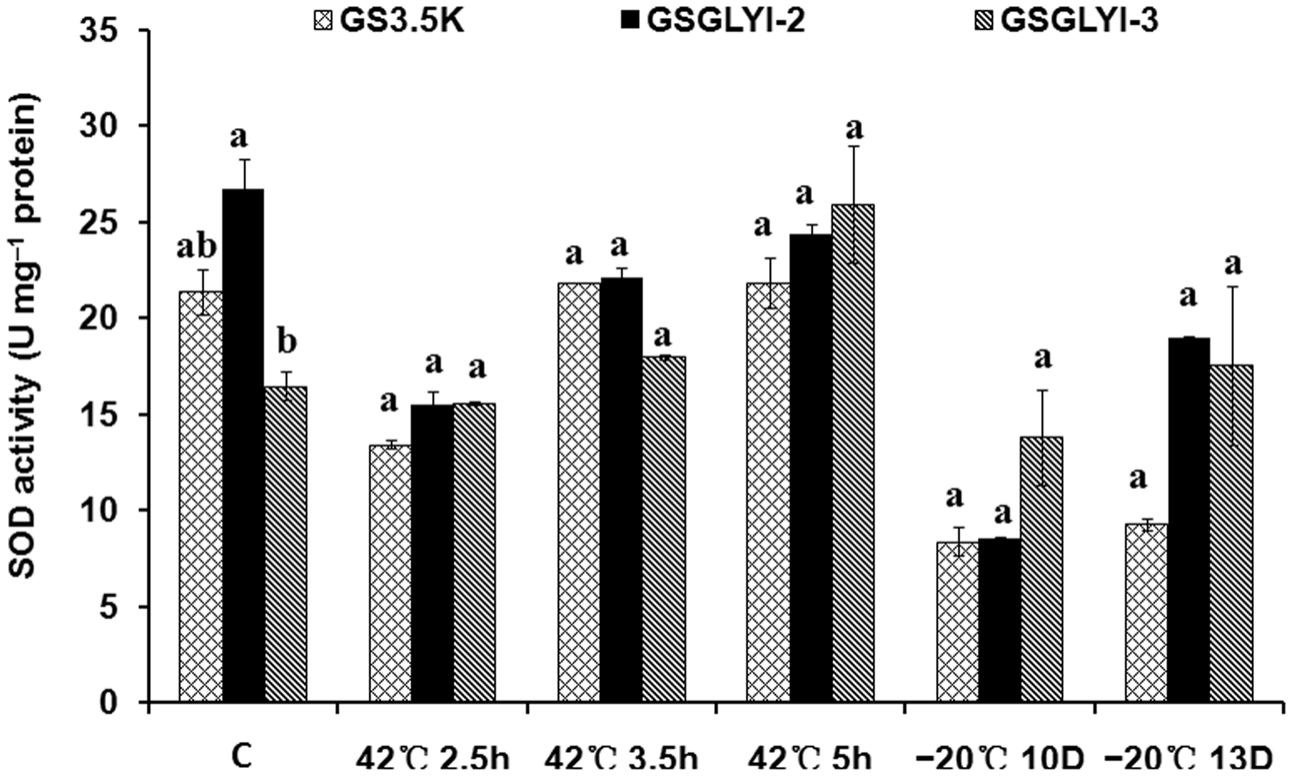
**Superoxide dismutase activity in transformed *P. pastoris* cells under temperature stress treatments.** The lowercase letters indicate significant differences among the transformants under the same treatment (Tukey’s multiple comparisons test, *P* < 0.05). Variants possessing the same letter are not statistically significant. C, control.

To further explore the physiological traits by which the GSGLYI-3 cells exhibited enhanced tolerance to temperature stress, the activity of the antioxidant enzyme SOD was examined (**Figure 5**). SOD showed different activity patterns among the different cells under heat and cold stress. SOD activity in GSGLYI-3, GSGLYI-2 and GS3.5K cells was similar under heat and cold stress treatments. However, compared to the same cells under control conditions, SOD activity was decreased by 8.6% in GSGLYI-2 cells treated at 42°C for 5 h, but was increased by 2.1 and 57.1% in GS3.5K and GSGLYI-3 cells, respectively (**Figure 5**). After treatment at –20°C for 13 D, SOD activity was decreased by 56.5 and 28.9% in GS3.5K and GSGLYI-2 cells, respectively, but was increased by 6.4% in GSGLYI-3 cells relative to the levels in the corresponding control-treated cells (**Figure 5**).

## Discussion

In the present study, two *BnGLYI* genes were isolated from *B. napus* seeds via a proteomic analysis of thermotolerant (3382) and thermosensitive (2682) seeds. The coding sequences of *BnGLYI-3* and *BnGLYI-2* were cloned and characterized from the thermotolerant and thermosensitive *B. napus* cultivars, respectively. A previous study found that the *BjGLYI* sequence showed significant homology with other GLYI sequences from plant and microbial species at the nucleotide and AA levels (Veena et al., 1999). In our study, the AA sequences of BnGLYI exhibited up to 79–98% identity to the AA sequences of GLYI from other seed plants. The *GLYI* coding sequence encodes for a 185 AA protein that contains zinc and GSH binding domains similar to the other reported GLYI proteins. Most interestingly, we found only two variations (Pro/Ala at position 9 and Ala/Val at position 93) between the AA sequences of BnGLYI-2 and BnGLYI-3.

There are 11 members of the *GLYI* family in both *Arabidopsis* and rice (Mustafiz et al., 2011). *B. napus* is an allotetraploid that may harbor many *GLYI* genes (at least two). After the release of the *B. napus* genomic sequence data^5^, BLAST searches were performed against the *B. napus* database. The results showed that the top hit was the assembled CDS of accession no. GSBRNA2T00156270001 using the AA sequence of BjGLYI (the peptides detected by MALDI-TOF MS analysis) as a query. When the corresponding CDS sequence was used as a query, the top hit was also GSBRNA2T00156270001. These results indicated that we cloned the gene identified by two-dimensional gel analysis. To fully understand the relationship between the two BnGLYI variants and resistance to temperature stress, we expressed the two *BnGLYI* genes in *P. pastoris*, a relatively simple system, and assessed their resistance to stresses *in vitro*. The results indicated that cells expressing the *BnGLYI-3* gene were significantly resistant to heat and cold stress compared with cells harboring an empty vector or expressing *BnGLYI-2.* Thus, these results demonstrated that the Pro9Ala and Ala93Vla variants may be responsible for the different levels of resistance to thermal stress between the two transformants.

Although glyoxalases have been highly conserved throughout evolution, they exhibit a significant structural variation in terms of active site position and number (Mustafiz et al., 2014). In the case of yeast (possessing a monomeric molecular structure), the AA residue mutation E163Q/E318Q of GLYI substantially reduced its activity, and this result confirmed that the yeast GLYI contains two active sites (Frickel et al., 2001). Human glyoxalase I is a homodimeric protein (Ridderström et al., 1998). It has been proposed that residue 172 in the human enzyme, a glutamate, acts as the base for catalysis of the reaction (Ridderström et al., 1998; Cameron et al., 1999). Others have argued that residue 157 of human GLYI has no direct role in catalysis, but is rather involved in forming the substrate-binding site (Ridderström et al., 1997). Met157 was changed into Ala, His or Glu, which resulted in either sharply decreased activity or the same high specific activity as wild-type GLYI (Ridderström et al., 1997). In the present study, of the two variations, only residue 93 was in the metal binding site, according to the structure of GLYI. Converting Ala93 to Val93 caused the cells to lose their resistance to heat and cold stress. This variation might lead to structural changes that could be related to the catalytic activity of GLYI.

The glyoxalase system, a ubiquitous detoxification system, is considered to play an important role in the removal of MG, a cytotoxic compound whose abundance increases during various stresses (Yadav et al., 2005a). In plants, this pathway is considered to be associated with tolerance to various abiotic stresses. GLYI in *B. juncea* was reported to confer resistance to salt stress (Veena et al., 1999; Singla-Pareek et al., 2003). In onions, upregulation of GLYI activity was observed in response to various stresses (Hossain and Fujita, 2009). In addition to MG detoxification, the physiological significance of the glyoxalase system is the maintenance of redox homeostasis via the regeneration of GSH. In our study, overexpressing *BnGLYI-3* gene from the heat-tolerant *B. napus* cultivar in yeast conferred resistance to heat and cold stress and significantly elevated GLYI activity compared to the control. These results suggest the protective role of enhanced BnGLYI-3 expression. Moreover, reduced GLYI activity results in MG accumulation (Shamsi et al., 2000). Thus, we speculated that an increase in activity of BnGLYI-3 could protect plants from heat and cold damage to a certain extent.

Furthermore, abiotic stress can stimulate ROS production and induce oxidative stress in plants (Hoque et al., 2012). During oxidative stress, the activity of antioxidant enzymes such as SOD generally increases in plants, and this elevated antioxidant enzyme activity correlates with increased stress tolerance (Gill and Tuteja, 2010). In a previous study, overexpression of a *B. campestris* HSP70 in tobacco clearly conferred enhanced tolerance to heat stress, and transgenic tobacco plants exhibited significantly higher SOD activity than wild-type plants. These results indicated that elevated SOD activity helps to alleviate the damage to the membrane system caused by heat stress (Wang et al., 2015). In our study, compared with the same cells under control condition, the SOD activity of GSGLYI-2 cells was decreased, but that of GSGLYI-3 cells was significantly increased after treatment at 42°C for 5 h. After treatment at –20°C for 13 D, GS3.5K and GSGLYI-2 cells exhibited decreased SOD activity, but GSGLYI-3 cells exhibited increased SOD activity. These results suggested thatGSGLYI-3 cells exhibit less accumulation of ROS and more effective growth than GS3.5K and GSGLYI-2 cells under temperature stress.

## Conclusion

We have identified a *BnGLYI* gene, and the overexpression of a variant of this gene in yeast conferred thermotolerance. BnGLYI may also possess a similar function in *B. napus* seeds. Additionally, the BnGLYI activity level may be affected by the AA variation at position 93. The results of our study may shed light on the mechanisms by which plants cope with heat stress.

## Author Contributions

Designed the experiments: GY and XW. Conducted the experiments: GY, XL, and XX. Analyzed the data: GY, XL, LW, and XX. Contributed materials/reagents/analysis tools: GY, XL, XX, GG, FL, JQ, JL, BC, KX, LW, and XW. Wrote the paper: GY.

## Conflict of Interest Statement

The authors declare that the research was conducted in the absence of any commercial or financial relationships that could be construed as a potential conflict of interest.

## Footnotes

1 http://www.matrixscience.com

2 http://www.lynnon.com.

3 http://www.ncbi.nlm.nih.gov/Structure/cdd/wrpsb.cgi.

4 http://pfam.sanger.ac.uk/

5 http://BrassicaDB.org/brad/

## Abbreviations

ROS: reactive oxygen species
GSH: glutathione (reduced)
MG: methylglyoxal
GLYI: glyoxalase I
GLYII: glyoxalase II
*BjGLYI*: *B. juncea GLYI* gene
*BnGLYI*: *B. napus GLYI* gene.
