# Supplementary material for: Identification and Characterization of a Glyoxalase I Gene in a Rapeseed Cultivar with Seed Thermotolerance
Source: Front Plant Sci. 2016 Feb 16;7:150. doi: 10.3389/fpls.2016.00150 (PMC4754733; doi:10.3389/fpls.2016.00150)
Supplement: Supplementary file 1 [file Data_Sheet_1.DOC]

**Supplemental Material**

**Title:** Identification and characterization of a *glyoxalase I* gene in a rapeseed cultivar with seed thermotolerance

**Running title:** *Glyoxalase I* gene in thermotolerant rapeseed

**Authors:** Gui-xin Yan Xiao-dan Lv Gui-zhen Gao Feng Li Jiangwei Qiao Jun Li Kun Xu Bi-yun Chen Li-min Wang Xin Xiao Xiao-ming Wu *

**Affiliation:** Oil Crops Research Institute of the Chinese Academy of Agricultural Sciences, Key Laboratory of Biology and Genetic Improvement of Oil Crops, Ministry of Agriculture, Wuhan 430062, P. R. China

***Corresponding author**: Xiaoming Wu, Oil Crops Research Institute of the Chinese Academy of Agricultural Sciences, Key Laboratory of Biology and Genetic Improvement of Oil Crops, Ministry of Agriculture, Wuhan 430062, P. R. China

Tel: +86-27-86812906; Fax: +86-27-86812906

E-mail: [wuxm@oilcrops.cn](mailto:wuxm@oilcrops.cn)

**Competing Interests:** The authors have declared that no competing interests exist.


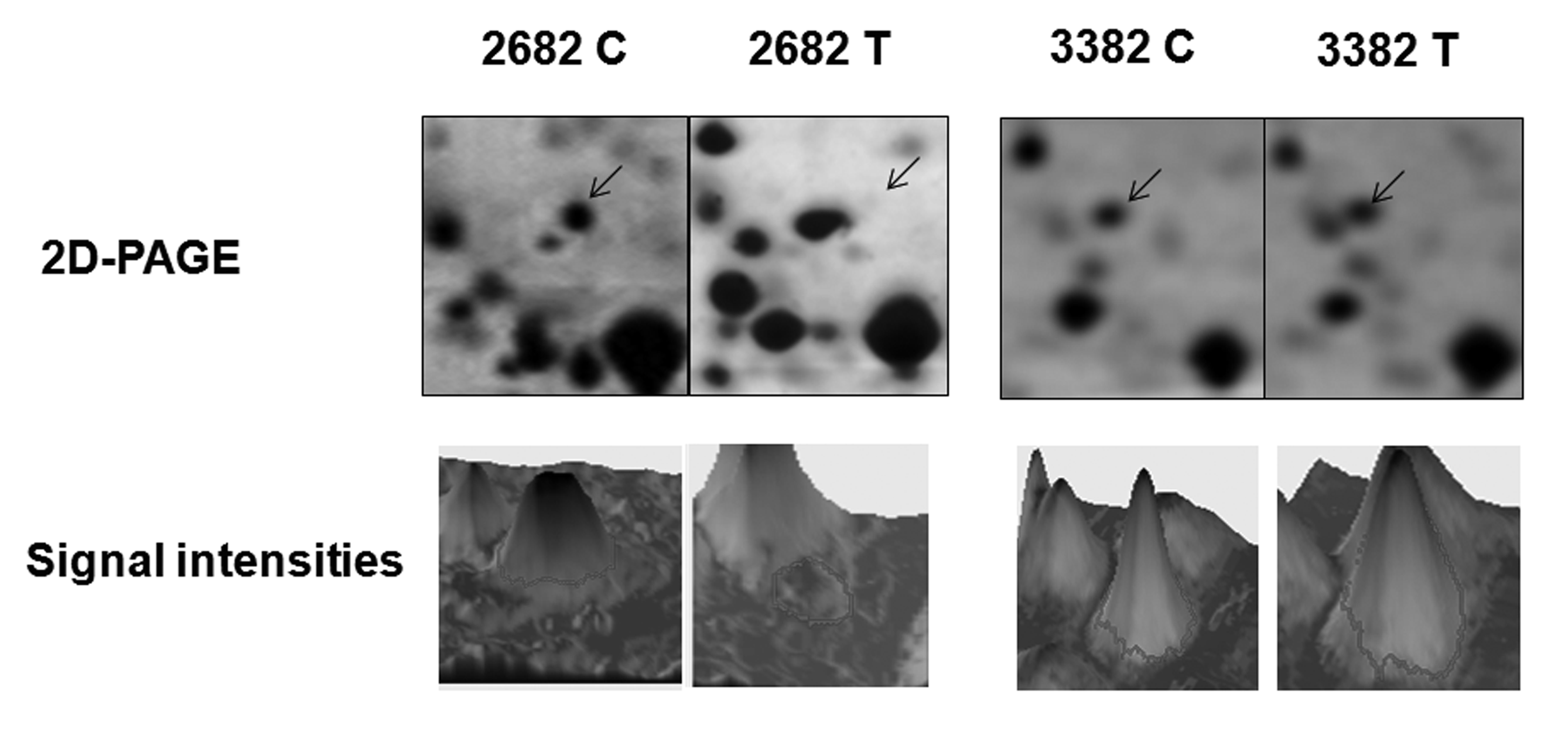


**Supplemental figure S1 A heat stress-responsive protein was identified by two-dimensional gel electrophoresis analysis.**

The quantified signal intensity of the protein spot from each image is shown in A (2682) and B (3382).

C: control, T: treatment


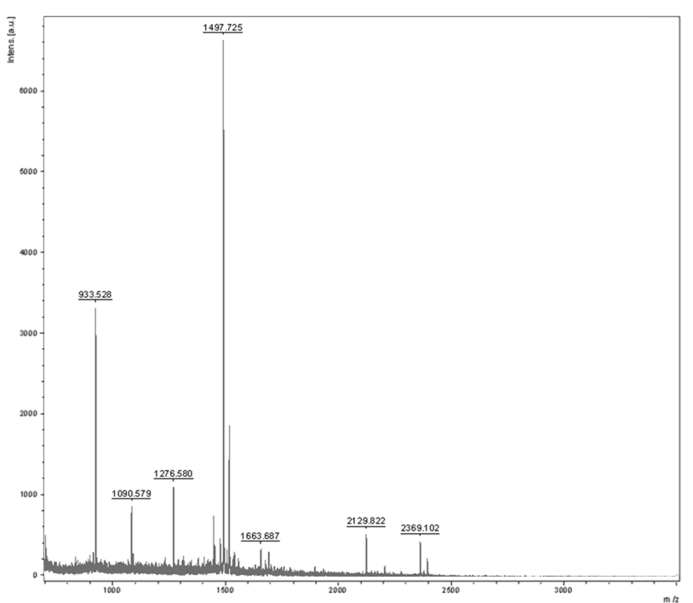


**Supplemental Figure S2 Identification of the selected protein based on its MALDI-TOF peptide mass map.** Search results with the corresponding mass data revealed the target protein as GLYI (*B. juncea*). MASCOT score: 117; mass values matched: 9.


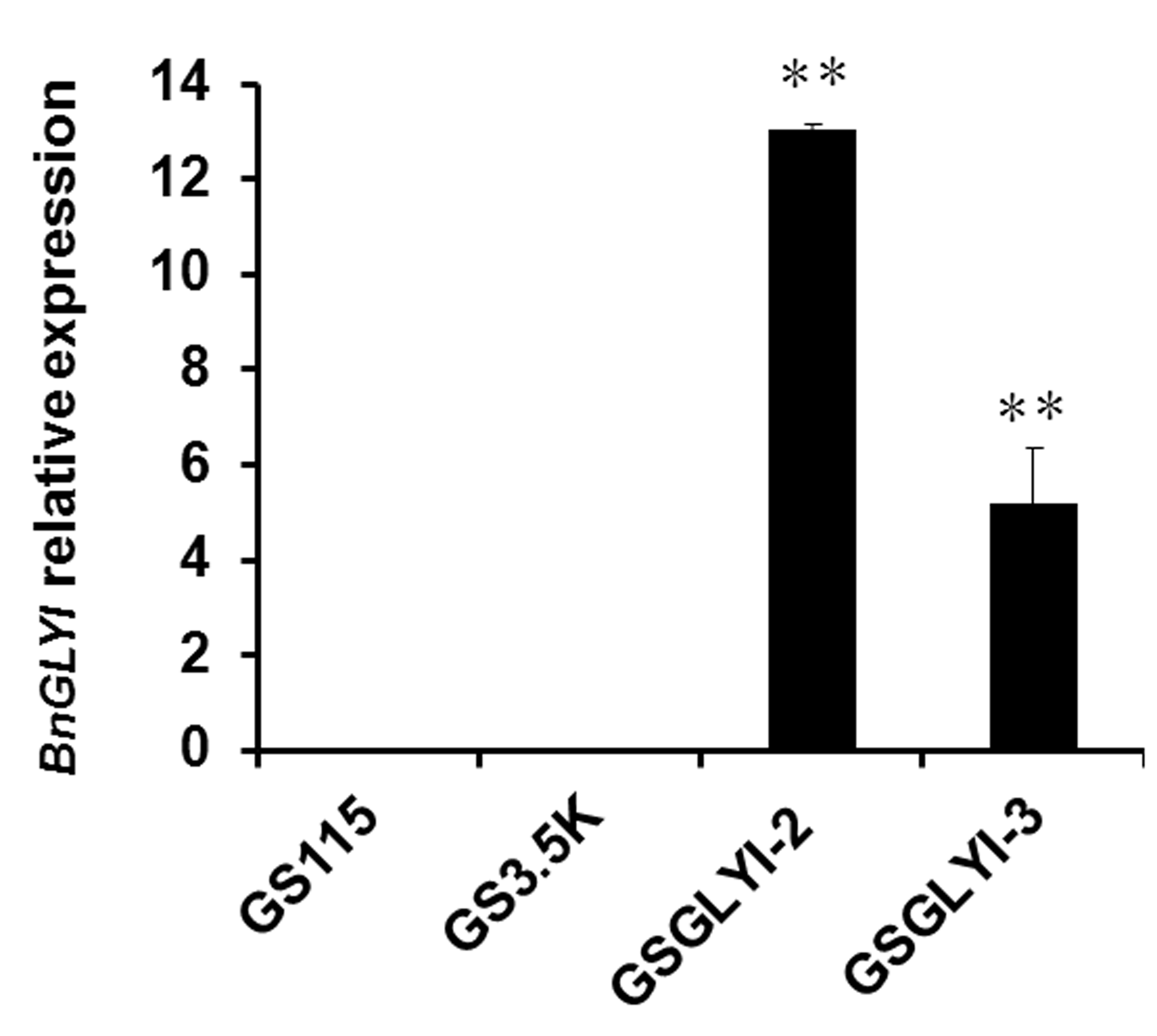


**Supplemental figure S3 qRT-PCR showing the expression of *BnGLYI* in** **wild-type and transgenic cells.**

Asterisks denote a significant difference compared with GS115 or GS3.5K cells based on Student’s t test; **P < 0.01.

The bars represent the means (three replicates) ± standard deviation.
